# Supplementary material for: Performance of Marmoset Monkeys as Embryo Donors Is Reflected by Different Stress-Related Parameters
Source: Animals (Basel). 2022 Sep 14;12(18):2414. doi: 10.3390/ani12182414 (PMC9494952; doi:10.3390/ani12182414)
Supplement: Supplementary file 1 [file animals-12-02414-s001.zip › animals-1787537-supplementary.pdf]

Supplemental Table S1. Performance data per animal in the short-term and long-term groups.

Group	Animal Number	Age in Months by Study Inclusion	Anti-Müllerian Hormone in ng/ml	Body Weight in g	Embryo Retrieval Rate (ERR)	Baseline Cortisol 0 Weeks after Anesthesia in µg/ml	Cortisol 3 Weeks after Anesthesia in µg/ml	Cortisol 6 Weeks after Anesthesia in µg/ml
Short-term	14604	35	11.56	359	0.45	1.68	1.2	1.12
	13845	56		346	0.64	2.00	1.18	1.4
	14234	48		442	0.95	4.44	3.26	1.2
	14963	45		395	0.33			
	13779	37		347	0.28			
	14025	55	9.04	369	0.06			
	14420	65		412	0.33	2.84	1.48	2.06
	14402	49		391	0.38			
	13794	37	10.28	349	0.63			
	13836	25		384	0.85			
	16228	122		419	0.83	1.54	1.08	1.16
	13802	30	8.92	449	0.87			
	13861	35		442	0.46			
	14745	63		392	0.17	1.66	1.08	1.28
	13806	48		351	0.22			
	14474	30	11.2	431	0.27			
	13681	63		344	0.22	1.94	1.4	1.24
	12944	77		463	0.64			
	14976	35		382	0	2.02	1.24	1.84
	14837	48		425	0	1.68	1.1	1.4
	15020	43		366		2.4	2.04	1.74
	14324	53		383		2.54	1.84	1.52
	14370	38		458		1.92	1.44	1.22
Long-term	13455	29	22.36	416	0.91			
	13637	29		431	1.28			
	14196	22		402	0.86			
	14457	28	16.88	396	0.53			
	14637	44		461	1.00	1.7	1.16	0.92
	14456	32	14.56	436	1.09	1.88		1.58
	14178	50		426	0.96	1.5		1.56
	15230	27	18.00	366	0.94	1.44	1.66	1.68
	12177	88		426	0.85	1.6	1.52	1.02
	14488	36	29.24	402	1.15	1.8	1.18	1.18
	13805	15		343	0.81			
	14204	43		391	0.96	1.3	1.4	

Supplemental Table S2. Number of included animals per figure.

Figure	Name of Figure	STG: Number of Animals	LTG: Number of Animals	STG + LTG Sum	Years of Use STG/LTG
1a	Ovarian cycle			1	
1b	Liver enzymes	2	2	4	
2	Embryo retrieval rates	20	12	32	0–2.5/2.5–5.0
3	Successful flushes	20	12	32	0–2.5/2.5–5.0
4	Anti-Müllerian Hormone	5	5	10	
5	Cortisol	12	7	19	
6	Weight	5	4	9	

Supplemental Table S3. Pathologic events.

Animal Number	Pathologic Event	Temporary Exclusion in Days	Fully Exclusion from Analysis
13,455	Hematoma in leg after blood sampling	4	no
13,637	Hematoma in leg after blood sampling	5	no
13,637	Metatarsus fraction	18	no
13,805	Loose paresis of foot	11	no
14,178	diarrhea	2	no
14,402	Nephritis in autopsy	0	no
14,963	Elevation of liver enzymes	0	no
14,420	Cheek swelling with tooth extraction	9	no
14,370	Ovary cyst		yes
13,823	Loose paralysis of leg		yes
13,710	Sudden death		yes
14,709	Anesthesia incident with death		yes
14,687	Chronic diarrhea		yes
13,902	Ovary cyst		yes
15,020	Osteolysis in lower jaw		yes
14,324	Leg aneurysm		yes
